# The impact of service-learning methodology on business schools’ students worldwide: A systematic literature review

**DOI:** 10.1371/journal.pone.0244389

**Published:** 2020-12-21

**Authors:** Marta Marco-Gardoqui, Almudena Eizaguirre, María García-Feijoo

**Affiliations:** Management Department, University of Deusto (Deusto Business School), Bilbao, Spain; Universitat Jaume I, SPAIN

## Abstract

The purpose of this research is to perform a systematic review of the literature published on the benefits of applying the service-learning methodology with business students. Several works and studies identify significant contributions that are important to the student’s profile; however, a systematic review of the benefits derived from the methodology application in the profile previously described has not yet been conducted. The main objective of this study is to gather, identify, and classify evidence from 32 studies conducted across global business schools regarding the benefits of service-learning in university students. The applied methodology follows the “Preferred Reporting Items for Systematic Reviews and Meta-Analyses” statement for systematic reviews. The studies were extracted from the World of Science, Scopus, and Educational Resource Information Center databases and were analyzed according to the suggested objectives. The suggested systematic review provides a rigorous analysis of academic literature on the benefits of service learning in the student’s personal, social, academic, and civic environment. A theoretical framework that combines all gathered benefits from the 32 studies has been suggested in these pages, by means of grouping these benefits into four categories considering prior research on the academic, personal, social, and citizenship outcomes. Several quantitative, qualitative, and mixed studies support the benefits of this methodology, focusing on the student’s profile development. We found that students enjoy plenty of outcomes after participating in the service-learning experience, thus, the most frequent outcome is greater social engagement. With more than 4,000 students involved in the total analyzed studies, we present a list of ranked outcomes that reflects and supports the strength of the methodology. Through this study, institutions as well as teachers may be aware of the potential present in this methodology. Our study also suggested a framework for university coordinators to act.

## Introduction, background and literature review

Business schools contemporarily face the challenge of training people to be prepared for responding to not only the challenges of organization management and administration but also manage social, ecological, and environmental challenges in the present context. Thus, higher education institutions have a pivotal role in providing future professionals with the knowledge, skills, and abilities that are required to deal with sustainability challenges identified in more global and complex societies [1, 2].

To some extent, organizations need managers who are able to fulfill the strategically suggested objectives presented by each one and must also face any potential external challenges. Furthermore, large, medium, and/or small companies have understood that the role played in society goes beyond satisfying their consumers’ short-term needs. The most successful companies have internalized and prioritized their sense of belonging to a greater community, in which it is of vital importance to combine short, medium, and long-term interests of all participants. Therefore, there is a need for business schools to prepare their students for behaving in an ethical and socially responsible manner [3, 4].

For such purposes, fostering new content, methodologies, learning experiences, ways to work collaboratively that allow students to develop critical thinking is necessary [5]. Likewise, to provide students with spaces where they can question their perspective of the world, the economy, and current society is necessary to try and develop a more honest and kinder society for all [6]. Recent studies have indicated that CEOs are in search of graduates with great analytical and strategic abilities, critical thinking, and leadership [7].

There are several methodologies that contribute to a students’ comprehensive training and among all of them, we have considered service-learning (SL) one of the best to fulfill this purpose [8]. SL is a methodology that is gaining popularity due to the link between theoretical learning and practical application, additionally providing value to the community under the social responsibility framework [9].

Various research demonstrate that SL experiences cause beneficial effects in students’ learning results [10–12]. For example, SL experiences increase students’ civic responsibility, their sense of effectiveness, and their professional and interpersonal skills [13, 14].

However, research conducted to study the benefits of SL have not always been performed in a rigorous scientific manner. Additionally, the scope of application and methodologies used have been quite varied [15].

Aware of the value of using the SL methodology in business schools, we present below a systematic review on this subject. A systematic review of the literature published in three important databases (Scopus, Web of Science, and Education Resources Information Center) has been performed to understand the benefits of the use of this methodology in the field of management studies. Therefore, the purpose of this review is to learn the impact of the SL application in academic, personal, and social training of students in business schools worldwide.

## Methods

The research conducted in this study presents a systematic review following the guide by Gough, Oliver and Thomas [16] (2012) and the methodology developed by the EPPI Center. Borrego, Foster, and Froyd state that this is one of the three systematic review outlines most used in multidisciplinary fields, such as business [17].

In addition, the verification list of the Joanna Briggs Institute (JBI) and the PRISMA recommendations have been considered with the aim of offering transparency, validity, and replicability to the study. A qualitative type of systematic (meaning that several of the main studies considered are qualitative) and configurative review (focusing on the status and the nature of the concepts found rather than on their thoroughness) has been applied.

### Research question

A common way to formulate research questions in systematic studies is by following the PICO framework developed by the National Institute for Health and Clinical Excellence. The acronym PICO stands for P (patient, problem, or population), I (intervention), C (comparison, control, or comparator), and O (outcomes) [18, 19]. According to this, our research question is the following: what are the benefits (outcome) of using SL methodology (intervention) with business students (population)? Therefore, the purpose is to learn the benefits of applying SL methodology in course subjects for students’ personal, academic, and social development. Studies were identified through a systematic search of key words, and they were also designed using the PICO strategy, with which, as aforementioned, three key elements are identified: population, intervention, and results.

### Search strategy

The following aspects have been considered to conduct the systematic review. First, we have considered articles published in international scientific journals, without considering a particular start date and up to October 2019. Second, keeping in mind its significance in the educational field, the following databases have been analyzed: WOS, Scopus, and ERIC. Third, studies were identified through a systematic search with key words: “service learning,” “service-learning,” “business school,” “business education,” and “management education” as shown in Table 1. Aiming to improve the search, the key words were combined. Publications comprising the search criteria in the title, keywords, and/or the abstract (depending on the search allowed in each database used) were included.

**Table 1:** Research question and keywords formulated with the PICO strategy.

Research question	What are the benefits (outcome) of using SL methodology (intervention) with business students (population)?		
PICO	[1] Population	[2] Intervention	[2] Outcome
	Business education	Service-learning	Benefits
	Management education	Service learning	
	Business School		

With the purpose of making the study easier to replicate, as suggested by [20] Ferreira Gonzalez, Urrutia, and Alonso-Coello, we present the search and terms used in the databases used in the following table (Table 2).

**Table 2:** Search terms and databases.

Search terms	Scopus	WOS	ERIC
• Service learning OR service-learning AND ○ education ○ Management education ○ Business school	TITLE ABS KEY	TITLE TOPIC	TITLE DESCRIPTOR ABSTRACT TITLE

#### Inclusion and exclusion criteria

With the purpose of designing the inclusion and exclusion criteria of the studies in a complete and unbiased manner, the PICO strategy was also used [21]. We have excluded from the study the following articles: (1) those reporting on the SL methodology application but in educational environments different from business; (2) those addressing the use of other methodologies for students’ training different from SL; (3) those in which specific empirical studies have not been conducted on the benefits of SL in students. Therefore, the studies’ inclusion criteria are the following: (1) those that refer to business schools; (2) those that use SL; (3) experience-based studies.

The documents that met the inclusion criteria across several stages were considered for data analysis and extraction, which represent a total of 32 studies. It is important to mention that the selection of studies was performed independently by three researchers, aiming to increase reliability and safety in the process [16].

#### Trial flow/selection process

**Fig 1:**
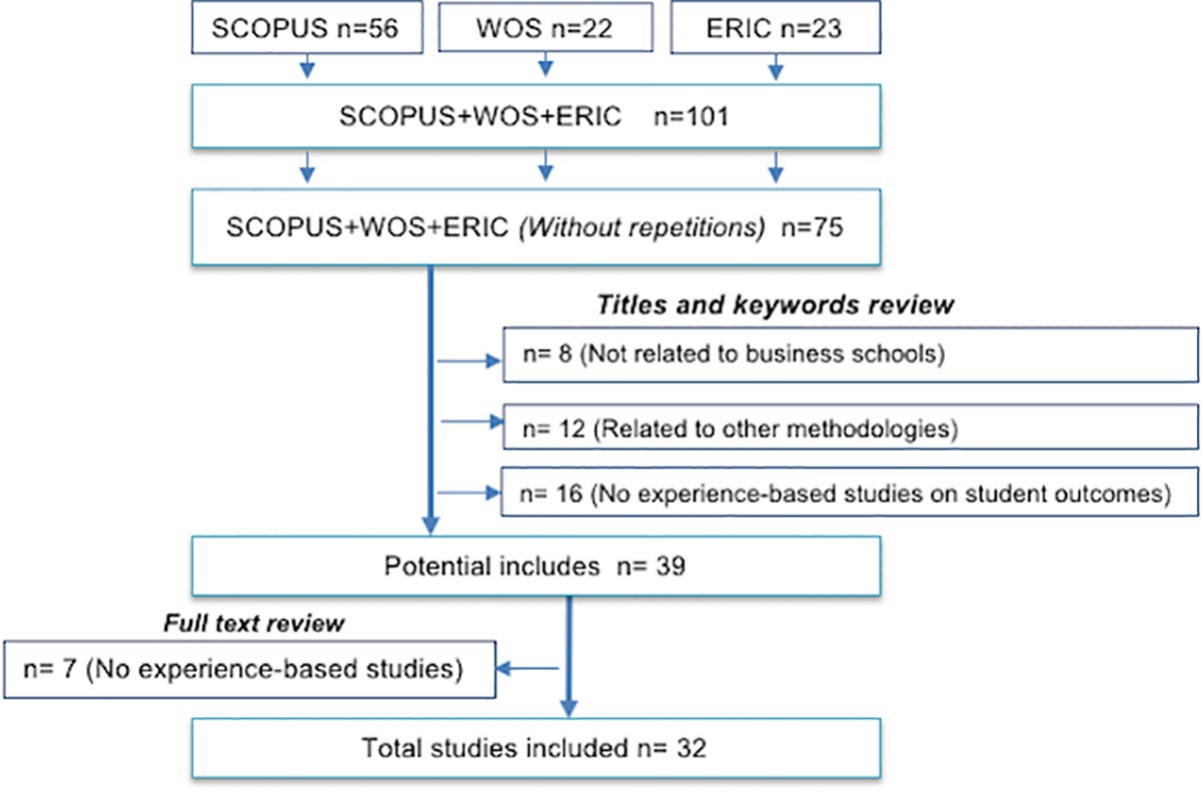
Flow diagram to show the process of study election.

All literature published in conferences, congresses, symposia, seminars, colloquia, workshops, and/or conventions was ruled out during the search. During the initial search, 101 documents were identified to be analyzed: 56 articles in Scopus, 22 in WOS, and 23 in ERIC. After the search was conducted, all the articles found were exported to Mendeley and all duplicate documents (n = 26) were eliminated. Thus, 75 articles remained to be analyzed. Fig 1 illustrates a flow diagram regarding the selection process of the studies.

Based on the 75 articles, we proceeded to review titles, keywords and abstracts. According to the first exclusion criterion, 8 documents were ruled out, 12 according to the second criterion, and 16 with the third criterion.

Second, a complete review of the texts was conducted, and 7 documents were ruled out based on the third criteria, thus leaving a total of 32 documents to be analyzed. Researchers unanimously agreed on the selection process of the articles, which may be the result of the use of simple and concrete selection criteria. Furthermore, the selection divided into stages enabled the cooperation among researchers to refine and clarify the criteria in a repetitive manner. Results are shown in the Fig 1.

The quality of the selected studies was assessed according to the 10 key control questions list suggested by the Joanna Briggs Institute (JBI) for systematic reviews [22]. The results of the 32 studies assessment are presented in Table 3.

**Table 3:** Quality of studies.

• *Q1*: *Is there congruity between the stated philosophical perspective and the research methodology*? • *Q2*: *And between the research methodology and the research questions or objectives*? • *Q3*: *And between the research methodology and the methods used to collect data*? • *Q4*: *And between the research methodology and the representation and analysis of data*? • *Q5*: *And between the research methodology and the interpretation of results*? • *Q6*: *Is there a statement locating the researcher culturally or theoretically*? • *Q7*: *Is the influence of the researcher on the research (or vice-versa) addressed*? • *Q8*: *Are participant and their voices adequately represented*? • *Q9*: *Is the research ethical according to the current criteria or does it even include ethical approval by an appropriate body*? • *Q10*: *Do the conclusions drawn in the research report flow from the analysis/interpretation of the data*?											
Authorship	References	Q1	Q2	Q3	Q4	Q5	Q6	Q7	Q8	Q9	Q10
Blewitt, Parsons, Shane (**2018**)	[23]	YES	YES	YES	YES	YES	YES	NO	NO	YES	YES
Braunsberger, Flamm (**2013**)	[24]	YES	YES	YES	YES	YES	YES	NO	YES	NO	NO
Buchanan (**2014**)	[25]	YES	YES	-	YES	YES	YES	YES	-	NO	YES
Carmichael, Rijamampianina (**2008**)	[26]	YES	YES	NO	YES	YES	NO	NO	NO	YES	YES
Chen, Snell, Wu (**2018**)	[27]	YES	YES	YES	YES	YES	YES	YES	YES	YES	YES
Coffey, Wang (**2006**)	[28]	YES	YES	YES	NO	YES	NO	NO	YES	YES	YES
Crutchfield (**2017**)	[29]	YES	NO	YES	NO	NO	-	-	YES	YES	-
Flannery, Pragman (**2008**)	[30]	YES	YES	YES	YES	YES	-	-	YES	YES	YES
Gallagher, McGorry (**2015**)	[31]	YES	YES	YES	YES	YES	-	YES	YES	YES	YES
Gerholz, Liszt, Klingsieck (**2018**)	[32]	YES	YES	YES	YES	YES	-	-	YES	YES	YES
Jordan, Schraeder (**2011**)	[33]	YES	YES	NO	NO	YES	NO	NO	NO	NO	YES
Killian, Lannon, Murray, Avram, Giralt, O'Riordan (**2019**)	[34]	YES	YES	YES	YES	YES	-	-	YES	YES	YES
Le, Raven, Chen (**2013**)	[35]	YES	YES	YES	YES	YES	YES	YES	YES	YES	YES
Levitt, Schriehans (**2010**)	[36]	YES	-	YES	YES	YES	-	-	YES	YES	YES
Madsen (**2004**)	[37]	YES	YES	YES	YES	YES	NO	NO	YES	YES	YES
Madsen, Turnbull (**2006**)	[38]	YES	YES	NO	NO	YES	-	-	NO	YES	YES
Metcalf (**2010**)	[39]	YES	YES	YES	YES	YES	-	YES	YES	YES	YES
Moorer (**2009**)	[40]	YES	YES	YES	YES	YES	NO	NO	YES	YES	YES
Mosakowski, Calic, Earley (**2013**)	[41]	-	-	YES	YES	YES	-	-	NO	YES	YES
Ngui, Voon, Lee (**2017**)	[42]	YES	YES	YES	YES	YES	NO	NO	YES	YES	YES
O’Brien, Wittmer, Ebrahimi (**2017**)	[43]	YES	YES	YES	YES	YES	-	-	YES	YES	YES
Palmer, Short (**2010**)	[44]	YES	YES	YES	-	-	YES	YES	YES	YES	YES
Peters, McHugh, Sendall (**2006**)	[45]	YES	YES	-	YES	YES	YES	YES	YES	YES	YES
Petrovskaya (**2019**)	[46]	YES	YES	YES	YES	YES	-	-	YES	YES	YES
Poon, Chan, Zhou (**2011**)	[47]	YES	-	YES	YES	YES	-	YES	YES	YES	YES
Sabbaghi, Cavanagh, Hipskind (**2013**)	[48]	YES	YES	YES	YES	YES	-	-	YES	YES	YES
Schneider (**2018**)	[49]	YES	-	YES	NO	YES	NO	-	YES	-	NO
Wang, Calvano (**2018**)	[50]	YES	YES	YES	YES	YES	NO	-	YES	YES	YES
Wickam (**2018**)	[51]	YES	YES	YES	YES	YES	-	-	YES	YES	YES
Wittmer (**2004**)	[52]	YES	YES	YES	YES	YES	-	YES	YES	YES	YES
Young, Karme (**2015**)	[53]	YES	-	-	NO	-	-	-	-	NO	YES
Yu (**2011**)	[54]	YES	YES	YES	-	YES	-	NO	YES	YES	YES

## Results

As already stated, 32 studies were analyzed in depth to respond to the previously suggested research question. The three researchers worked in parallel and in a coordinated action on an analytical framework that enabled the systematization and summarization of the most relevant information from the selected articles. First, a general description of the articles was considered (authors’ affiliation, origin, subject/area of expertise in which the methodology is applied, and study’s date of publication); second, the methodology used in the study (nature of the research, type of study design, tool to gather information, samples, and scales used); third, the types of results (benefits on students); and finally, conclusions of each study. Each researcher analyzed the results independently and the main ideas were compared with the purpose of reaching a consensus on the main conclusions of the systematic review.

In order to build a reference framework on the benefits obtained by management students when experiencing SL during the university training, the descriptors of the main studies are detailed below.

### Studies’ descriptors

Of all 32 reviewed studies, it is important to mention that they have all been written in English. Regarding their authors’ affiliation, they are mostly from American universities. SL is acknowledged as a legitimate part of business education, as shown by the curricula of institutions such as the University of Michigan, Wharton, Wisconsin-Madison, Bentley College, and Loyola University of Chicago [52], which accounts for the amount of studies conducted in the United States.

Of the total analyzed studies, 23 of them are from American universities and one of them is co-authored by the United States and China. Of the remaining studies, we note that two were coauthored by teachers of the same departments in Hong Kong and Canada and from Australia and Finland. Regarding the other 7 studies, they are from Ireland, Germany, Hong Kong, Malaysia, Philippines, Russia, and South Africa.

Regarding the journals where these analyzed studies have been published, they are quite varied, with the Journal for Education for Business having the most articles published, with a total of 6, as can be observed in Table 4.

**Table 4:** Journal of publication.

Journal (in alphabetical order)	No. of documents	References	Indexing and Impact factor 2019
Academy of Management Learning & Education	2	Mosakowski, Calic, Earley (**2013**) [41]; Chen, Snell, Wu (**2018**) [27]	• Scopus (SJR Impact factor: 1.89) • WoS (JCR Impact factor: 4.058)
Active Learning in Higher Education	1	Gerholz, Liszt, Klingsieck (**2018**) [32]	• Scopus (SJR Impact factor: 2.07) • WoS (JCR Impact factor: 3.118)
American Journal of Business Education	1	Moorer (**2009**) [40]	• ERIC
Business Horizons	1	Schneider (**2018**) [49]	• Scopus (SJR Impact factor: 1.4) • WoS (JCR Impact factor: 3.444)
Development and Learning in Organizations	1	Jordan, Schraeder (**2011**) [33]	• Scopus (SJR Impact factor: 0.16)
Education + Training	2	Young, Karme (**2015**)[53]; Ngui, Voon, Lee (**2017**) [42]	• Scopus (SJR Impact factor: 0.75) • WoS (JCR Impact factor: 1.791)
International Atlantic Economic Society	1	Gallagher, McGorry (**2015**) [31]	• ERIC
International Journal of Teaching and Learning in Higher Education	1	Peters, McHugh, Sendall (**2006**) [45]	• ERIC
Journal of Business Ethics	3	Flannery, Pragman (**2008**) [30]; Wittmer (**2004**) [52]; Sabbaghi, Cavanagh, Hipskind (**2013**) [48]	• Scopus (SJR Impact factor: 1.97) • WoS (JCR Impact factor: 4.141)
Journal of Education for Business	6	Madsen (**2004**) [37]; Coffey, Wang (**2006**) [28]; Crutchfield (**2017**) [29]; Blewitt, Parsons, Shane (**2018**) [23]; Wang, Calvano (**2018**) [50]; Le, Raven, Chen (**2013**) [35];	• Scopus (SJR Impact factor: 0.44)
Journal of Higher Education Outreach and Engagement	1	Wickam (**2018**) [51]	• Scopus (SJR Impact factor: 0.38)
Journal of Instructional Pedagogies	1	Levitt, Schriehans (**2010**) [36]	• ERIC
Journal of International Education in Business	2	Buchanan (**2014**) [25]; Poon, Chan, Zhou (**2011)** [47]	• Scopus (SJR Impact factor: 0.3)
Journal of Management Education	2	Madsen, Turnbull (**2006**) [38]; O’Brien, Wittmer, Ebrahimi (**2017**) [43]	• Scopus (SJR Impact factor: 0.66) • WoS (JCR Impact factor: 2.354)
Journal of Marketing Education	1	Metcalf (**2010**) [39]	• Scopus (SJR Impact factor: 1.01)
Journal on Excellence in College Teaching	1	Palmer, Short (**2010**) [44]	• ERIC
New Horizons in Education	1	Yu (**2011**) [54]	• Not indexed at this moment (SJR Impact factor 2016: 0.101)
On the Horizon	1	Petrovskaya (**2019**) [46]	• Scopus (SJR Impact factor: 0.17)
Problems and Perspectives in Management	1	Carmichael, Rijamampianina (**2008**) [26]	• Scopus (SJR Impact factor: 0.2)
The International Journal of Management Education	1	Killian, Lannon, Murray, Avram, Giralt, O'Riordan (**2019**) [34]	• ERIC
Voluntas	1	Braunsberger, Flamm (**2013**) [24]	• Scopus (SJR Impact factor: 0.81) • WoS (JCR Impact factor: 1.538)

Regarding the SL area of knowledge, Table 5 shows the subjects analyzed. We found a higher percentage of studies on the Marketing and MBA subjects.

**Table 5:** Subjects/areas of knowledge in which the studies are based.

Course Content (in alphabetical order)	Studies
Accounting	Yu (**2011**) [54]
Business Communications	Blewitt, Parsons, Shane (**2018**) [23]
Business Ethics	O’Brien, Wittmer, Ebrahimi (**2017**) [43]
Finance	Buchanan (**2014**) [25]
Human resources	Madsen (**2004**) [37]; Madsen, Turnbull (**2006**) [38]; Peters, McHugh, Sendall (**2006**) [45]
Marketing; International Marketing; Marketing Research and Strategic Marketing; Fundamentals of Marketing and Consumer Behaviour; Marketing for social change	Metcalf (**2010**) [39]; Braunsberger, Flamm (**2013**) [24]; Crutchfield (**2017**) [29]; Wang, Calvano (**2018**) [50]; Schneider (**2018)** [49]; Killian, Lannon, Murray, Avram, Giralt, O'Riordan (**2019**) [34]
Management	Flannery, Pragman (**2008**) [30]; Levitt and Schriehans (**2010**) [36]
Marketing and Finance	Gallagher, McGorry (**2015**) [31]
MBA	Wittmer (**2004**) [52]; Coffey, Wang (**2006**) [28]; Carmichael and Rijamampianina (**2008**) [26]; Sabbaghi, Cavanagh, Hipskind (**2013**) [48]; Young, Karme (**2015**) [53]; Chen Snell, Wu (**2018**) [27]; Wickman (**2018**) [51]
Social Innovation	Ngui, Voon, Lee (**2017**) [42]
Subjects not specified	Moorer (**2009**) [40]; Palmer, Short (**2010**) [44]; Jordan, Schraeder (**2011**) [33]; Poon, Chan, Zhou (**2011**) [47]; Mosakowski, Calic, Earley (**2013**) [41]; Le, Raven, Chen (**2013**) [35]; Petrovskaya (**2019**) [46], Gerholz, Liszt, Klingsieck (**2018**) [32].

From the 32 analyzed studies, only 7 specifically state the participants’ year in the degree program. Among them, the SL methodology has been applied to the second, third, and final year of the study course, which seems logical given that toward the end of their course, the participants can contribute to the community the most.

**Fig 2:**
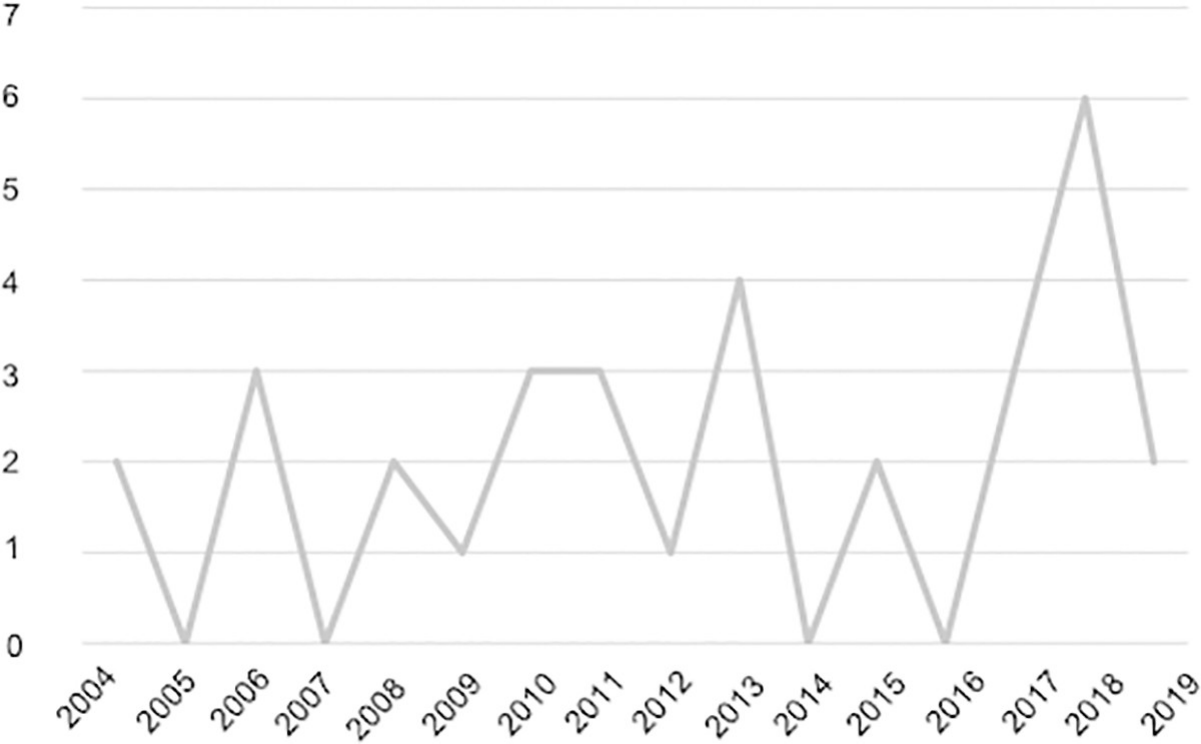
Number of publications per year.

Regarding the publication date, the first published studies on the benefits of applying the SL methodology in business students dates back to 2004, and since then, studies have been continuously published to date (Fig 2).

It is important to mention that the trend in terms of the number of publications on the subject in recent years has increased, with 2018 as the year when there were maximum publications. However, we cannot conclude that publications declined in 2019, since our search of studies used October of the same year as a limit.

#### Studies’ methodology

**Fig 3:**
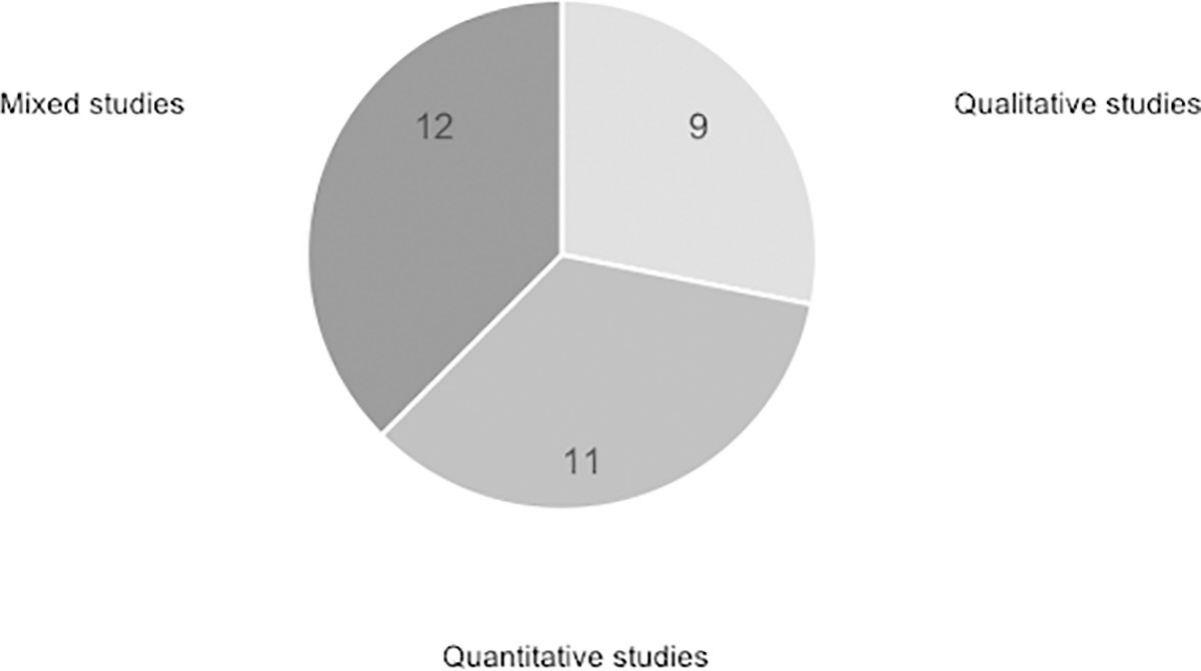
Percentage of different types of studies.

From the 32 analyzed studies and considering the methodology used in each one of them, 9 articles used qualitative techniques, 11 used quantitative techniques, and 12 of them used mixed techniques, as illustrated in Fig 3.

Regarding the tools used for the research, they varied according to the nature of the research. Thus, in qualitative studies, written reflections and in-depth interviews are used as pivotal instruments for gathering information. In quantitative studies surveys are the most employed instrument. In this case, face-to-face surveys prevail over online surveys. Finally, mixed studies combine the aforementioned instruments.

Regarding the studies’ research design, in most cases (20), descriptive cross-sectional simple or multiple research was conducted, interviewing the students only once at the end of the SL experience. In rest of the studies (12), longitudinal descriptive research was conducted. In these studies, the students are asked a series of questions before, during, and after the SL experience. Longitudinal descriptive research is more common in studies that combine mixed techniques (qualitative and quantitative) and less common in merely qualitative studies.

The type of study is distinguished based on the student samples used for the analysis of each article, with the mean of participants per qualitative study being 48 students (median = 21, with 3 studies not specifying the sample), the mean of participants per quantitative studies being 228 students (median = 105), and the mean per mixed study being 106 students (median = 88, considering that 3 of the studies do not specify the sample). Below, we carry out a more detailed analysis of the three types of studies: qualitative, quantitative, and mixed. First, Table 6 gathers the characteristics of qualitative studies.

**Table 6:** Features of the qualitative studies.

Research instrument	Research design	Author(s)	Sample size
Reflection paper	Multiple cross-sectional	Crutchfield (**2017**) [29]	20–40 students
		Levitt, Schriehans (**2010**) [36]	120 students
	Single cross-sectional	Ngui, Voon, Lee (**2017**) [42]	107 students
		Buchanan (**2014**) [25]	*(Not specified)*
	Longitudinal	Coffey, Wang (**2006**) [28]	*(Not specified)*
		Young, Karme (**2015**) [53]	*(Not specified)*
In-depth interviews	Single cross-sectional	Madsen, Turnbull (**2006**) [38]	10 students
		Mosakowski, Calic, Earley (**2013**) [41]	9 students
	Longitudinal	Madsen (**2004**) [37]	12 students

Regarding the used research tools, we notice that reflection papers are most common, followed by in-depth interviews. With regard to the research design, with the information provided by the authors, there are more cross-sectional studies (26) than longitudinal ones (4), in addition to a causal research and a study in which the design type used for the analysis is not specified.

According to the study’s samples, it can be noted that they are naturally smaller than the ones used in quantitative studies [55], since the aim of the former is to explore, and the latter’s aim is to describe or measure. This is also the case for the documents revised in our analysis, since in qualitative studies, as the study progresses in gathering data, this does not imply that more information is being produced [56].

Table 7 shows the main characteristics of quantitative studies are provided.

**Table 7:** Features of the quantitative studies.

Research instrument	Research design	Author(s)	Sample size
Survey	Multiple cross-sectional	Peters, McHugh, Sendall (**2006**) [45]	45 students
		Carmichael, Rijamampianina (**2008**) [26]	72 students
		Moorer (**2009**) [40]	120 students; 90 students
		Palmer, Short (**2010**) [44]	1530 students
		Poon, Chan, Zhou (**2011**) [47]	120 students
		Yu (**2011**) [54]	187 students
		Braunsberger, Flamm (**2013**) [24]	19 students
		Blewitt, Parsons, Shane (**2018**) [23]	50 students
Online survey		Wang, Calvano (**2018**) [50]	70 students; 104 students
		Gallagher, McGorry (**2015**) [31]	185 students
		Flannery, Pragman (**2008**) [30]	123 students; 91 students

The most used research tool is face-to-face surveys, and only in a few cases, the survey is conducted online. Regarding the research design, all of these are multiple cross-sectional studies.

Third, in Table 8 we present the characteristics of mixed studies which combine qualitative and quantitative methodologies.

**Table 8:** Features of the mixed studies.

Research instrument	Research design	Author (s)	Sample size
Survey and reflection paper	Single cross-sectional	Wittmer (**2004**) [52]	71+147 students
		Metcalf (**2010**) [39]	19+72 students
		Sabbaghi, Cavanagh, Hipskind (**2013**) [48]	88 students
Reflection paper		Jordan, Schraeder (**2011**) [33]	*(Not specified)*
Survey and phone interview		Wickam (**2018**) [51]	*(Not specified)*
Survey and reflection paper	Multiple cross-sectional	Chen, Snell, Wu (**2018**) [27]	275 students
		Petrovskaya (**2019**) [46]	152 students
		Le, Raven, Chen (**2013**) [35]	17 students
Survey and interview		Killian, Lannon, Murray, Avram, Giralt, O'Riordan (**2019**) [34]	*(Not specified)*
Reflection paper	Longitudinal	O’Brien, Wittmer, Ebrahimi (2017) [43]	215 students
		Schneider (2018) [49]	16 students
Survey and reflection paper	Causal research	Gerholz, Liszt, Klingsieck (**2018**) [32]	36 students

In most cases, a prior questionnaire and a reflection paper by the student is combined after the SL experience. Regarding the research design, most of them are cross-sectional, with no longitudinal methodologies being applied.

#### Rubrics and scales used in the studies

The most common scales to measure SL benefits are self-reporting measures [57]. There is no generally accepted scale to measure the benefits but there is one commonly used: the SELEB (*SErvice LEarning Benefits)* scale, developed by Toncar, Reid, Burns, Anderson, Nguyen in 2006 [58]. The scale consists of 12 items grouped in 4 dimensions: practical skills, interpersonal skills, citizenship, and personal responsibility skills. This scale has been adapted and modified according to different situations as we will see in the different analyzed studies. For example, in one of the articles, the latest version of the scale that includes 20 different items and a five-point Likert scale have been used to measure, among other aspects, leadership, communication skills, and sense of community. In the rest of the analyzed documents, different measurement tools are used.

A classification of the different rubrics and scales used in the different studies (Table 9) for the research studies that are indicated are shown below.

**Table 9:** Scales used in the studies.

Type of study	Author(s)	Scales used
Quantitative studies	Gallagher, McGorry (**2015**) [31]	SELEB
	Moorer, Cleamon (**2009**) [40]	
	Flannery, Pragman (**2008**) [30]	Scale developed and validated: Weber, Weber, Sleeper, Schneider (2004) [60]
	Braunsberger., Flamm (**2012**) [24]	Weber, Glyptis (2000) [61]
	Wang, Calvano (**2018**) [50]	Furco (1996) SL assessment work [62]
		Young, Caudill, William (2008) [63]
		Geringer, Stratemeyer, Canton, Rice (2009) [64]
Mixed studies	Metcalf (**2010**) [39]	SELEB
	Gerholz, Liszt, Klingsieck (**2018**) [32]	Self-efficacy: Schwarzer, Jerusalem (1999) [65]
		Self-concept: Reinders, Wittek, (2009) [66]; Weber, Glyptis (2000) [61]
		Civic attitude: Mabry (1998) [67]
	Petrovskaya (**2019**) [46]	PVQ40 Portrait Values Questionnaire. Schwartz [68]
	O’Brien, Wittmer, Ebrahimi (**2017**) [43]	Ingol, Shapiro (2014) [59]
	Schneider (**2018**) [49]	The Other in the Self (IOS) scale

#### Studies’ content: SL outcomes and conclusions

In the analysis of the selected studies, we found that students enjoy plenty of outcomes after participating in the SL experience (Table 10). Thus, the most frequent outcome mentioned in the analyzed studies is greater social engagement acquired by the students. Second, and with the same number references, we found improved self-esteem (or trust in oneself) followed in third place by teamwork skills. Third, through SL application, students are able to achieve more meaningful learning.

**Table 10:** List of outcomes and number of times pointed out as a benefit.

Specific elements	N° studies
Greater commitment, justice, and social responsibility	11
Improved self-esteem and self-confidence	11
Improved teamwork skills	10
Meaningful learning	8
Enhanced leadership skills // Improved communication skills // Increased awareness of the nature of social issues and better knowledge of the actual society	7
Application of the theory to the "real world," transformational learning	5
Increased motivation // Improved problem-solving skills // Greater recognition of the diversity and intercultural understanding // Greater sense of solidarity through empathy and community engagement // Critical thinking	4
Presentation skills // Improved self-efficacy and self-management // Further reflection on their own privileges and values // Increased recognition of volunteering and perception of positive work model	3
Pride feeling	2

In addition to the previously mentioned results, we also observed a great presence of a more meaningful learning and enhanced leadership and communication skills.

**Fig 4:**
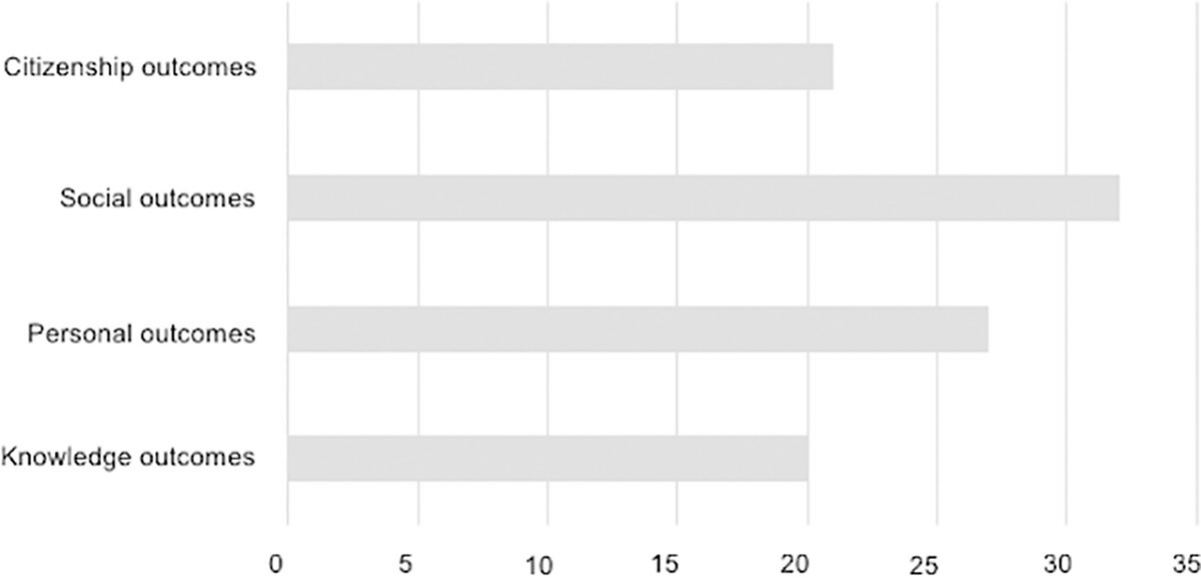
Number of studies that mention each category.

With regard to presenting and grouping the benefits reported by the use of the SL methodology in students who participate in it, we follow a classification based on the existing literature of studies on SL [69]. Thus, the following categories are used: a) knowledge outcomes (knowledge, cognitive results, academic motivation, and attitudes); b) personal outcomes (participants’ thoughts and feelings about themselves or their motives or values and their well-being); c) social outcomes (ability to interact or work with others, to understand or tolerate diversity, beliefs or attitudes towards others); and d) citizenship outcomes (responsible, participatory, and just citizenship). Fig 4 summarizes the number of studies that mention each benefit category.

Social outcomes are the most mentioned category in terms of benefits, followed by personal outcomes, citizenship outcomes, and knowledge outcomes.

**Fig 5:**
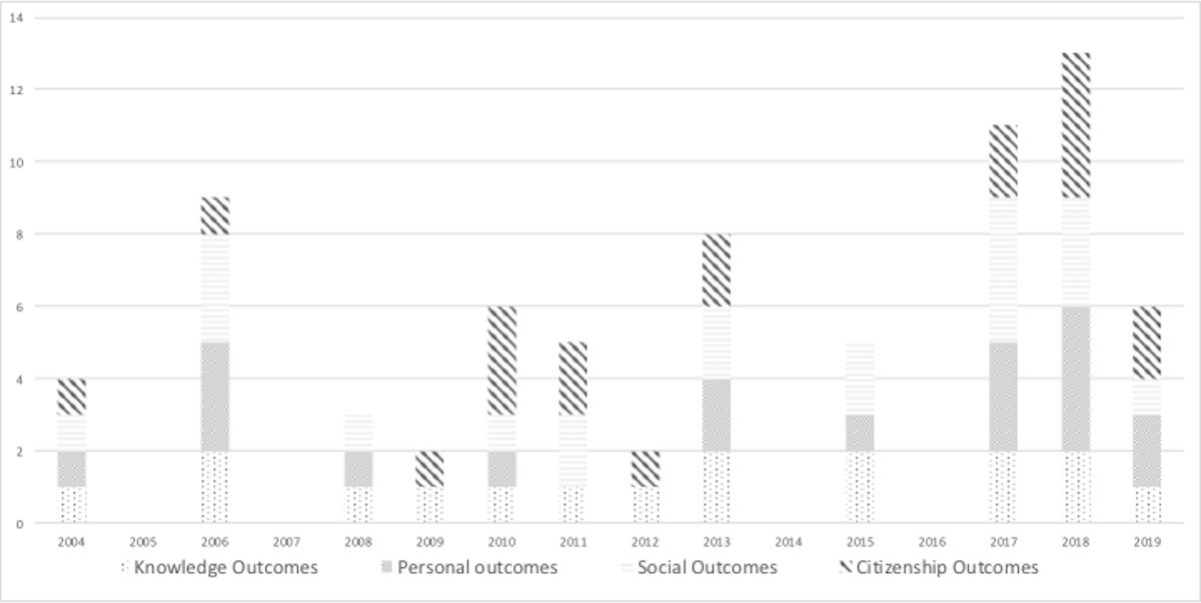
Development of the number of times each category is mentioned in the publications from 2004 to 2019.

**Fig 6:**
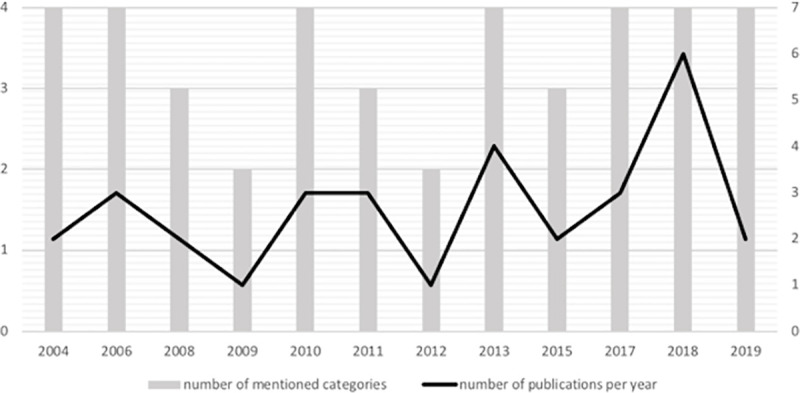
Comparison between the number of studies and the number of categories mentioned.

In the Figs 5 and 6, the different benefits obtained in each study, which are grouped in the 4 mentioned categories, are analyzed in terms of time.

Fig 5 illustrates the number of times each of the SL outcomes’ categories are mentioned within the time frame in which the studies have been published and considered for analysis. We found evidence of the four categories in studies published over seven years; however, there are six years in which the evidence gathered applies to only a few categories but not to all. Data referring to the number of publications per year have been compared with the number of references of each of the four categories to have a comprehensive understanding of the benefits obtained in the 32 analyzed studies (Fig 6). Thus, in 2004 and 2006, two and three articles were published respectively, in which the four categories were mentioned. In 2008 and 2009, three and two articles were published respectively, in which the different benefits were shown but failed to mention any of the four categories. In 2010 and 2013, three and four studies were published respectively, in which the four categories were mentioned. This was not the case in the following years (2011, 2012, and 2015). In the last three years (2017, 2018, and 2019), all publications referred to the four dimensions of SL benefits in a stable manner. As mentioned above, in the rest of the presented timeframe, the trend was not so steady. That is, there were alternate years in which evidence of the four categories was gathered and years in which only a few categories are addressed.

Within these four categories, the different studies gathered different specific elements. A summary of all the benefits compared in the 32 analyzed studies of this review are shown in Table 11.

**Table 11:** Outcomes and specific elements listed in the 32 studies analyzed.

Type of outcome	Specific elements	Number	Studies
Knowledge outcomes	Meaningful learning	8	Madsen (**2004**) [37]; Madsen, Turnbull (**2006**) [38]; Flannery, Pragman (**2008**) [30]; Yu (**2011**) [54]; Buchanan (**2013**) [25]; Gallagher, McGorry (**2015**) [31]; Crutchfield (**2017**) [29]; Ngui, Voon, Lee (**2017**) [42].
20 studies			
	Application of the theory to the "real world," transformational learning	5	Peters, McHugh, Sendall (**2006**) [45]; Moorer (**2009**) [40]; Young, Karme (**2015**) [53]; Wang, Calvano (**2018**) [50]; Wickam (**2018**) [51].
	Presentation skills	3	Crutchfield (**2017**) [65]; Wickam (**2018**) [51]; Killian (**2019**) [34].
	Increased motivation	4	Madsen, Turnbull (**2006**) [38]; Levitt, Schriehans (**2010**) [36]; Braunsberger, Flamm (**2012**) [24]; Le, Raven, Chen (**2013**) [35].
Personal outcomes	Improved self-esteem and self-confidence	11	Madsen (**2004**) [37]; Peters, McHugh, Sendall (**2006**) [45]; Metcalf (**2010**) [39]; Buchanan (**2013)** [25]; Le, Raven, Chen (**2013**) [35]; Crutchfield (**2017**) [29]; O’Brien, Wittmer, Ebrahimi (**2017**) [43]; Ngui, Voon, Lee (**2017**) [42]; Blewitt, Parsons, Shane (**2018**) [23]; Wang, Calvano (**2018**) [50]; Gerholz, Liszt, Klingsieck (**2018**) [32].
27 studies			
	Improved problem-solving skills	4	Peters, McHugh, Sendall (**2006**) [45]; Carmichael, Rijamampianina (**2008**) [26]; Wickam (**2018**) [51]; Killian, Lannon, Murray, Avram, Giralt, O´Riordan (**2019**) [34].
	Improved self-efficacy and self-management	3	Madsen (**2004**) [37]; Carmichael, Rijamampianina (**2008**) [26]; Gerholz, Liszt, Klingsieck (**2018**) [32].
	Critical thinking	4	Coffey, Wang (**2006**) [28]; Madsen, Turnbull (**2006)** [38]; Buchanan (**2013**) [25]; Killian, Lannon, Murray, Avram, Giralt, O´Riordan (**2019**) [34].
	Further reflection on their own privileges and values	3	Young, Karme (**2015**) [53]; Blewitt, Parsons, Shane (**2018**) [23]; Petrovskaya (**2019**) [46].
	Pride feeling	2	Madsen (**2004**) [37]; Killian, Lannon, Murray, Avram, Giralt, O´Riordan (**2019**) [34].
Social outcomes	Improved teamwork skills	10	Coffey, Wang (**2006)** [28]; Madsen, Turnbull (**2006**) [38]; Peters, McHugh, Sendall (**2006**) [45]; Carmichael, Rijamampianina (**2008**) [26]; Metcalf (**2010**) [39]; Yu (**2011**) [54]; Crutchfield (**2017**) [29]; Blewitt, Parsons, Shane (**2018**) [23]; Wickam (**2018**) [51]; Killian, Lannon, Murray, Avram, Giralt, O´Riordan (**2019**) [34].
32 studies			
	Enhanced leadership skills	7	Metcalf (**2010**) [39]; Jordan, Schraeder (**2011**) [33]; Sabbaghi, Cavanagh, Hipskind (**2013**) [48]; Gallagher, McGorry (**2015**) [31]; Ngui, Voon, Lee (**2017**) [42]; Schneider (**2018**) [49]; Killian, Lannon, Murray, Avram, Giralt, O´Riordan (**2019**) [34].
	Improved communication skills	7	Coffey, Wang (**2006**) [28]; Madsen, Turnbull (**2006**) [38]; Peters, McHugh, Sendall (**2006**) [45]; Metcalf (**2010**) [39]; Gallagher, McGorry (**2015**) [31]; Blewitt, Parsons, Shane (**2018**) [23]; Killian, Lannon, Murray, Avram, Giralt, O´Riordan (**2019**) [34].
	Greater recognition of the diversity and intercultural understanding	4	Mosakowski, Calic, Earley (**2013**) [41]; Young, Karme (**2015**) [53]; Ngui, Voon, Lee (**2017**) [42]; Schneider (**2018**) [49].
	Greater sense of solidarity through empathy and community engagement	4	Wittmer (**2004**) [52]; Coffey, Wang (**2006**) [28]; O’Brien, Wittmer, Ebrahimi (**2017**) [43]; Schneider (**2018**) [49].
Citizenship outcomes	Greater commitment, justice and social responsibility	11	Moorer (**2009**) [40]; Metcalf (**2010**) [39]; Levitt, Schriehans (**2010**) [36]; Palmer, Short (**2010**) [44]; Poon, Chan, Zhou (**2011**) [47]; Yu (**2011**) [54]; Sabbaghi, Cavanagh, Hipskind (**2013**) [48]; O’Brien, Wittmer, Ebrahimi (**2017**) [43]; Schneider (**2018**) [49]; Wang, Calvano (**2018**) [50]; Killian, Lannon, Murray, Avram, Giralt, O´Riordan (**2019**) [34].
21 studies			
	Increased awareness of the nature of social issues and better knowledge of the actual society	7	Coffey, Wang (**2006**) [28]; Braunsberger, Flamm (**2012**) [24]; Le, Raven, Chen (**2013**) [35]; Blewitt, Parsons, Shane (**2018**) [23]; Gerholz, Liszt, Klingsieck (**2018**) [32]; Killian, Lannon, Murray, Avram, Giralt, O´Riordan (**2019**) [34]; Petrovskaya (**2019**) [46].
	Increased recognition of volunteering and perception of positive work model	3	Wittmer (**2004**) [52]; Ngui, Voon, Lee (**2017**) [42]; Blewitt, Parsons, Shane (**2018**) [23].

**Fig 7:**
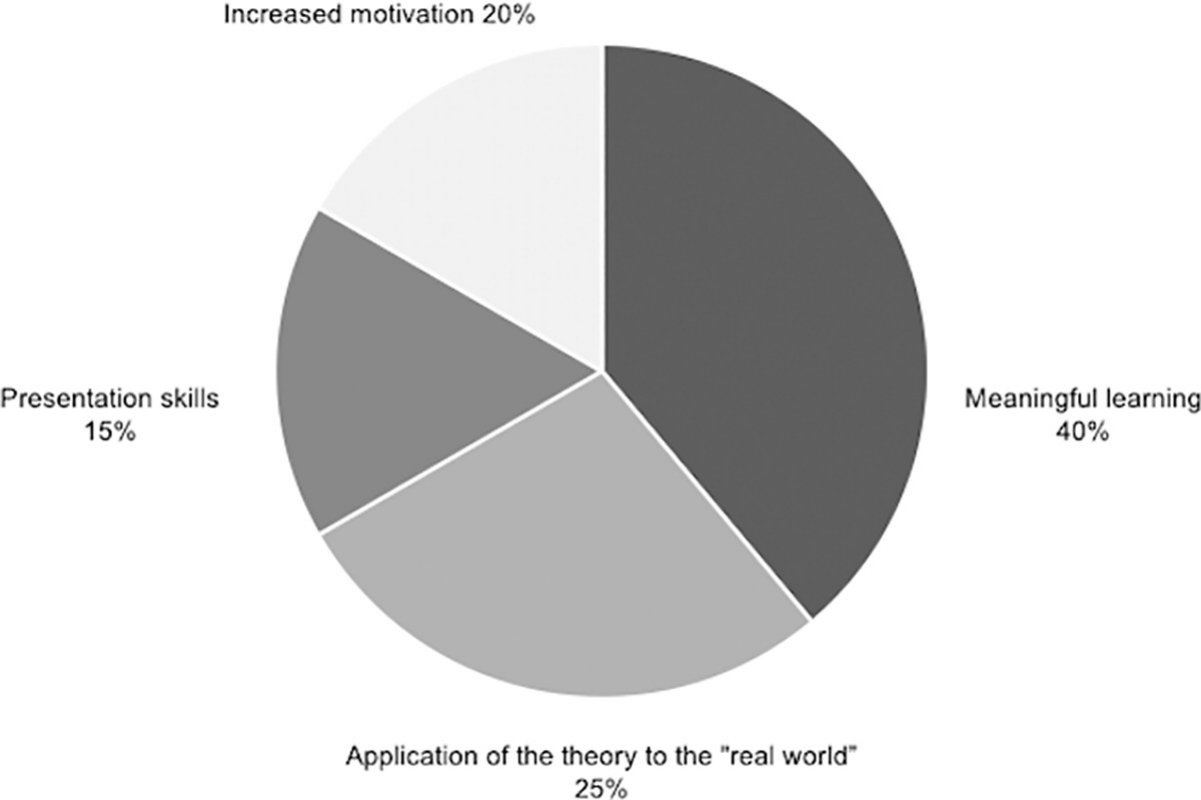
Percentage of studies that mention specific elements within knowledge outcome.

**Fig 8:**
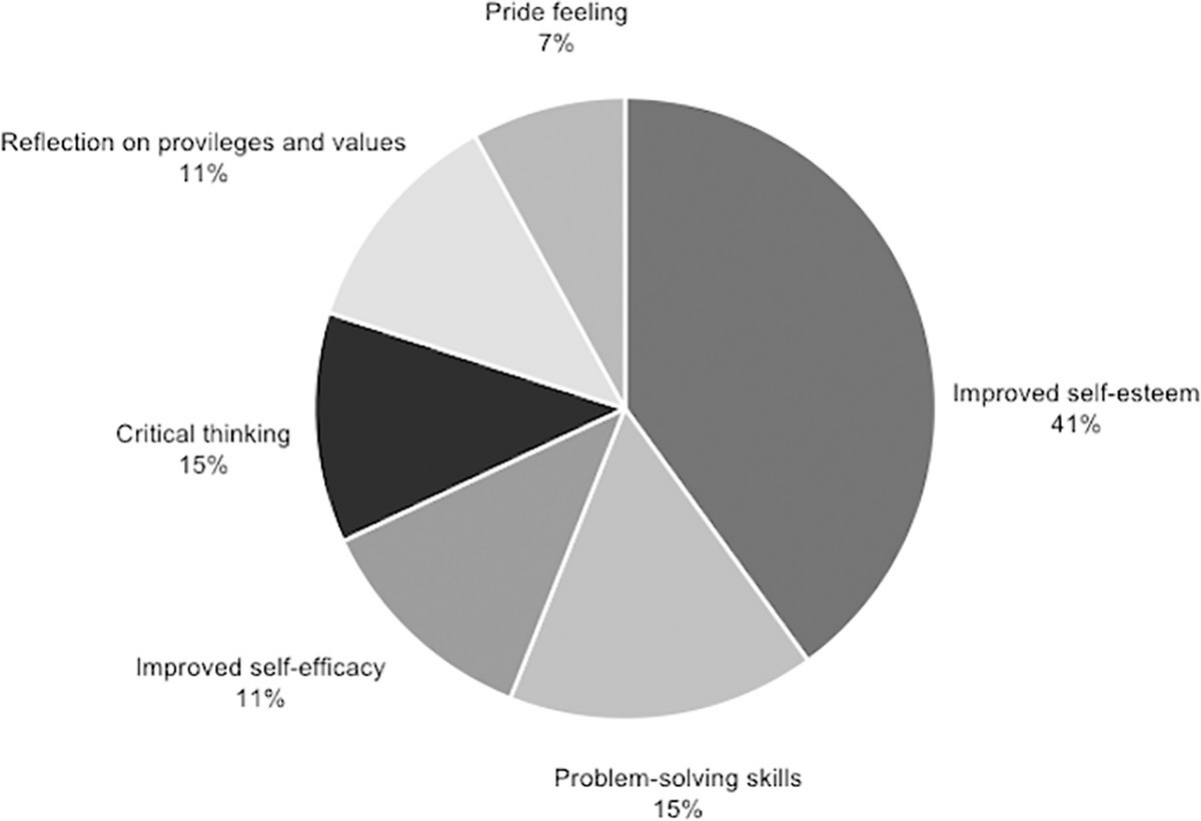
Percentage of studies that mention specific elements within personal outcome.

**Fig 9:**
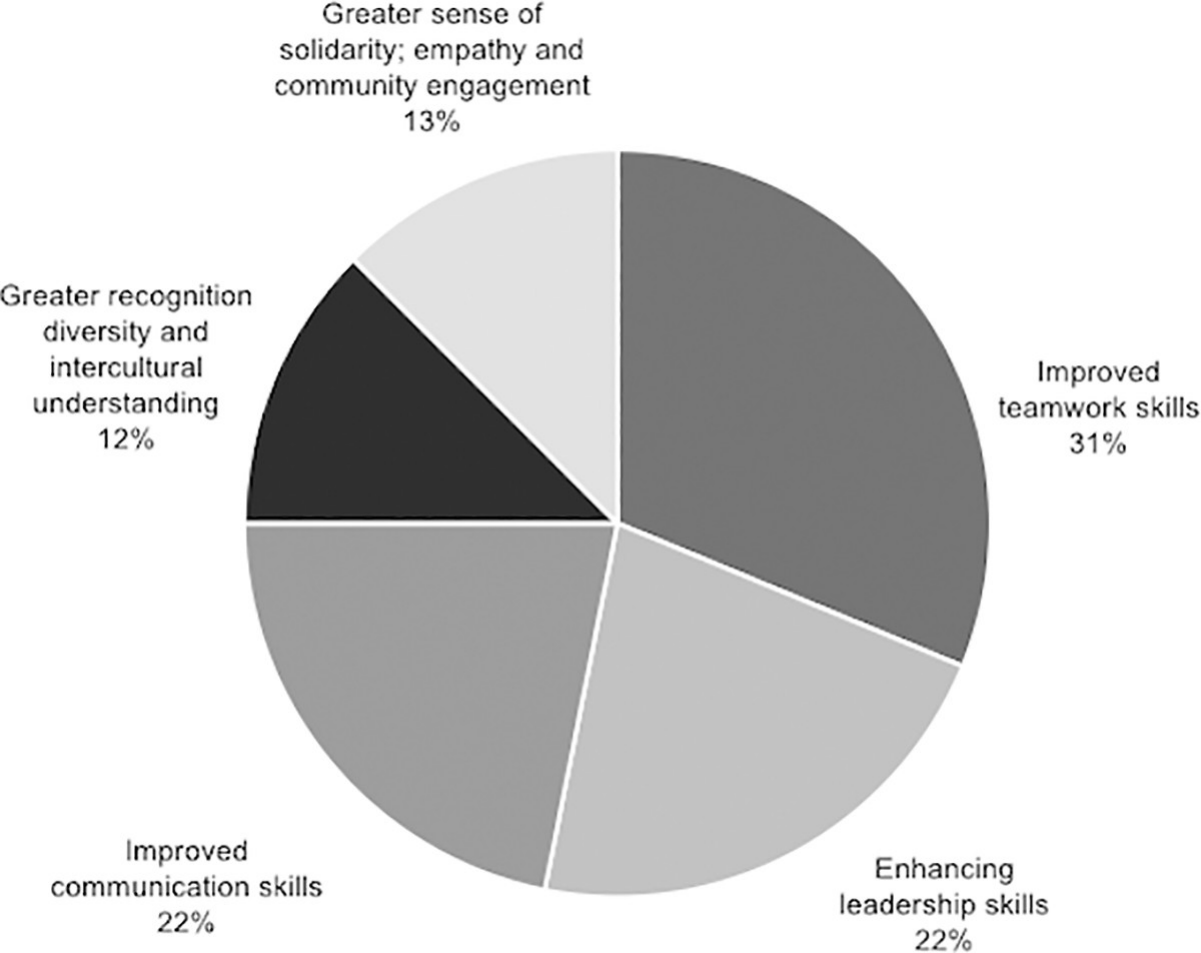
Percentage of studies that mention specific elements within social outcome.

**Fig 10:**
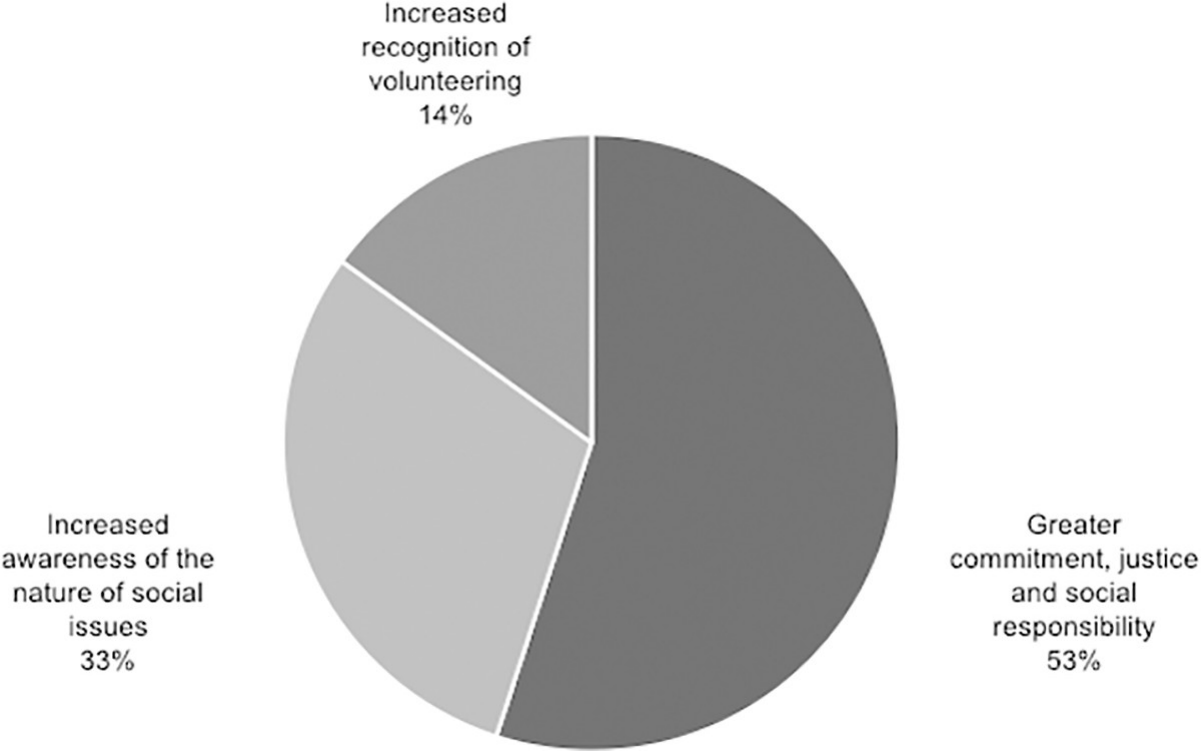
Percentage of studies that mention specific elements within citizenship outcome.

Important elements of each of the four categories in which the benefits of applying the methodology have been classified are visually represented in the Figs 7–10.

### Additional considerations

The 32 analyzed documents have different objectives and scope, and they use different methodologies. However, how applying the SL methodology in business schools can contribute to the students’ profile development is analyzed in all of the documents. With the purpose of answering our research question, to know the benefits of the application of the SL methodology in business school students, we can assert that the use of this methodology among management students reports some clear benefits at different levels.

Some influencing effects of SL benefits have been emphasized within the conducted analysis:

1. Gender elements. In a total of six articles, the gender variable is mentioned as a key element for the study’s analysis, as can be observed. Furthermore, in several future research proposals suggested by some authors, the suitability of classifying research by gender as well as by culture is presented.
2. Year in which the SL was experienced. As stated above, the grade level in which the SL is experienced seems to be an important element to obtain the previously mentioned benefits. Based on the methodology’s characteristics, it seems evident that students will take advantage of the experience once they have adapted and assimilated to the university’s functioning and gain maturity in their learning engagement.
3. The SL methodology is suitable for any academic discipline, although it is especially recommended for marketing courses [70–72]. This is mainly due to marketing’s interest in social causes [73] and, especially due to the fact that non-profit organizations tend to lack the abilities and resources to undertake or outsource marketing activities [71]. In this review, there are seven research studies that are exclusively conducted in marketing subjects, but we found plenty of applications in different areas such as management, finance, administration, and strategy.
4. E-service learning has the potential to transform both learning through community services as well as online learning, releasing SL learning from geographical limitations and providing online learning with a tool to foster engagement [74]. However, in most of the studies analyzed in the research, these experiences are conducted face-to-face, and online learning is seldom used.

## Discussion, limitations, and future research lines

After a thorough analysis of the 32 studies focused on the SL methodology that were conducted in this study, we observed that the results achieved are relevant for business schools at a global scale. First, it is important to emphasize that this topic is of great interest in the academic field. During the last few years, the trend for research on this subject has increased. Several quantitative, qualitative, and mixed studies support the benefits of this methodology, focusing on the student’s profile development. Furthermore, these benefits can be demonstrated not only in general higher education institutions but also in the specific area of business schools. A theoretical framework that combines all gathered benefits from the 32 studies has been suggested in these pages, by means of grouping these benefits into four categories considering prior research on the knowledge, personal, social, and citizenship outcomes [69]. In this sense, all the benefits derived from the application of the methodology in students may be included in some of these four categories. Although a sample size of a total of 4,202 students in this review is interesting in terms of the overall results obtained, one of the greatest limitations observed in some research is that it is conducted in small samples and specific contexts. We find that the research presented has different implications for practitioners and institutions. For many Higher Education Institutions and Business Schools, their participation in the Principles for Responsible Management Education (PRME) originated in the awareness of the role of business schools in contributing to the financial crisis [75]. and since part of the material traditionally taught by business schools can be seen as “amoral theories” that have actively freed students from any sense of moral responsibility” [76]. Therefore, it seems that the content taught by business schools has been a challenge in terms of social transformation for several years. This systematic review has proven that a way to actively influence this transformation is through classroom application of learning methodologies such as SL. By means of this methodology, social engagement and comprehension of the real needs of different communities are fostered, as well as providing positive working values for students. Through this study, institutions as well as teachers may be aware of the potential present in this methodology.

Furthermore, our study also suggested a framework for university coordinators to implement. The importance of distinguishing among four outcome categories that contribute to the SL experience is clearly stated in this work. According to these categories, academic coordinators may opt to choose any of these activities based on the professional-academic profile they pursue with the study program. The analyzed studies show the suitability to schedule SL activities not only to achieve academic but also personal, social, and citizenship outcomes. SL experiences are especially appropriate to foster greater commitment, justice, and social responsibility among students, as well as to foster self-esteem, self-confidence, and teamwork skills. In relation to the limitations and possible further research lines, a first group, composed of those found in the studies analyzed in the systematic review, and a second group, comprised of those inherent to our own research, can be distinguished.

Among the former, the following significant aspects can be stated:

- Most of the analyzed research studies have been conducted in American universities. Therefore, research is somehow geographically biased, and this should be considered when reviewing the conclusions. Completion of results with studies conducted in business schools from other countries and geographical areas is considered necessary to know the scope of impact that cultural and social specificities generate with regard to a different perspective in terms of the outcomes expected from SL experiences.
- Along the same line, most of the analyzed studies fail to mention the existing cultural differences between different students who participate in the experience, which can also be addressed in further research.
- The same occurs in reference to students’ gender. Although some of the analyzed studies consider them, this is not the case for other studies. Therefore, an important field of work in terms of acquiring a more real and reliable image of the methodology impact is opened.
- Regarding the measurements of the outcomes presented by the SL application and considering the existence of a specific and authenticated scale (SELEB), very few studies apply this. A more general application of this scale would be interesting to reach an integrated classification proposal of the resulting outcomes for applying the methodology.
- With regard to the research design, most of them are cross-sectional. In order to broaden conclusions, we believe longitudinal studies are required to assess the same students over an extended period of time, and thus observe the development and impact of the methodology application on the global profile of the student.

Limitations of the systematic review conducted are presented in a second group. These limitations create different future research lines for researchers interested in delving deeper into the role of SL in the development of business students’ profile. First, a search with key words in English was conducted, which left out articles published in another language for the review. It would be interesting to expand on this area in the future, as well as to perform systematic reviews of articles published in different languages, thus reducing the possibility for the studies to be biased by the predominance of American university studies, as previously mentioned. Second, the research question was focused on the benefits for students in applying the methodology. Therefore, articles dealing with the impact of the methodology on other parties involved (communities, teachers and universities, organizations, and companies) have been beyond the purview of the review. Further research considering other parties as objects of study would contribute to have a broader view on the methodology’s scope and potential.

Finally, types of business schools, as well as their affiliations, institutional values and mission, have not been differentiated in this study. Given the role of SL in the development of specific values and fostering self-knowledge and self-importance, it would be more useful to encourage research in relation to results and benefits achieved in different institutions (for example, religious versus secular).

## Supporting information

S1 Checklist PRISMA 2009 checklist. (DOC)
